# Suffering in silence: consequences of sexual violence within marriage among young women in Nepal

**DOI:** 10.1186/1471-2458-11-29

**Published:** 2011-01-12

**Authors:** Mahesh Puri, Jyotsna Tamang, Iqbal Shah

**Affiliations:** 1 Associate Director Centre for Research on Environment Health and Population Activities (CREHPA) Kathmandu, Nepal; 2 Research Associate Centre for Research on Environment Health and Population Activities (CREHPA) Kathmandu, Nepal; 3 Department of Reproductive Health and Research World Health Organization 1211 Geneva 27, Switzerland

## Abstract

**Background:**

Despite the grave consequences of sexual violence, and it's persistence both within and outside marriages, this subject has received relatively little attention from researchers, policy makers, and programme managers in Nepal. This paper explores the definition of sexual violence and its various forms and consequences as reported by young married women in Nepal. In addition, it describes the coping mechanisms used by young married women to avoid sexual violence perpetrated against them by their husbands.

**Methods:**

This paper analyzes data collected during the qualitative study on "Sexual violence among young couples in Nepal", conducted amongst two major ethnic groups - Brahmin/Chhetri and Tharu - between 2006 and 2007. The data is comprised of 39 free-lists and 15 in-depth case histories with married women aged 15-24 years. The average rank and Smith's Salience were calculated from the free-listing data. The thematic analysis approach was used for the analysis of information from the case histories.

**Results:**

Approximately three-quarters (74%) of the young women mentioned 'sex against one's wishes' as sexual violence within marriage (SVWM). Sixty-two percent of respondents described 'forced sex during illness' and 'forced sex after consumption of alcohol' as SVWM. About half of young women (48.7%) who participated in the free-listing exercise reported having experienced SVWM. The types of SVWM ranged from unwanted sexual touch to forced sex. Backache, headache, lower abdominal pain, vaginal bleeding and thoughts of suicide were the most commonly reported negative physical and psychological health consequences of SVWM. Women reported various coping strategies including 'trying to convince husbands', 'sleeping in a separate room', 'visiting maternal home', 'waking up the children', and 'using pretexts such as being ill or menstruating', to avoid SVWM. However, in most cases, women reported that these coping strategies were unsuccessful. Almost all women experiencing SVWM were socially isolated and did not turn to institutions, relatives or friends for advice and support.

**Conclusions:**

Sexual violence within marriage is common in Nepal. Findings provide circumstantial evidence of links between sexual violence and negative general and reproductive health outcomes for women. Various actions are required to prevent SVWM and provide immediate support to the victims.

## Background

Sexual violence within or outside marriage has been increasingly recognised as a major public health problem as well as a serious human rights abuse [1]. An expanding and persuasive body of evidence from diverse settings has documented the connection between sexual violence and reproductive and sexual health risks [1-7]. For example, coerced sex is associated with a range of gynaecological and reproductive health problems, including transmission of HIV and other sexually transmitted infections (STIs), unwanted pregnancy, vaginal bleeding or infection, fibroids, decreased sexual desire, genital irritation, pain during intercourse, chronic pelvic pain, and urinary tract infections [8-11]. Sexual violence can also lead indirectly to a variety of health problems, such as stress-induced psychological changes, substance abuse, and lack of fertility control and personal autonomy [12]. Despite this myriad of adverse outcomes, few studies on intimate partner violence and its consequences have been conducted in developing countries, particularly in South Asia. The experiences of young married women in particular remain largely unexplored.

A few studies in South Asia have explored the coping strategies used by young married women to avoid situations of high personal risk for sexual violence [13-18]. These studies revealed that young married women try to avoid unwanted sex with their husbands by threatening to scream, in order to endanger the husband's prestige, threatening to commit suicide, waking up young children, and feigning menstruation [13-18]. Alternatively, some women try to develop a greater intimacy with their husbands, communicate sexual desire, and participate more equally in sex-related decision making to avoid unwanted sexual experiences [17-19]. Though a few small-scale studies in Nepal have documented the extent and causes of sexual violence against women within marriage (SVWM), no previous study is known to have focussed on the consequences and coping strategies used by women to avoid SVWM and document the care and support seeking behaviours employed by these women in Nepal [14-16].

Nepal is a small landlocked country nestled in the foothills of the Himalayas. The People's Republic of China borders Nepal in the north and India borders in the east, south and west. Nepal has a diversity of cultures, climates, traditions and languages. The majority of the population (86%) lives in rural areas, with limited or no access to basic infrastructure or services [20]. Hinduism is the main religion of Nepal (81% of the population) [20].

In Nepal, patriarchal family structure is prevalent, with most women having little or no say about whom and when they marry, whether or not to bear children, and/or when and how many children to have [21]. Traditionally, boys and girls marry before the age of 18. Many girls in rural areas marry shortly after puberty, or sometimes even before. For the majority of Nepalese young women, sexual activity commences at an early age, mostly within marriage [21]. Despite the legal age of marriage being 18 years for both men and women with the consent of guardians, and 20 years without the consent of guardians, teenage marriage continues to be the norm among many ethnic groups. The median age at first marriage among women age 20-49 is 17.2 years [22]. Traditionally, marriages in Nepal are arranged by the respective families. Before arranging marriage, families consider a number of factors such as caste, religion, ethnicity and economic status, as well as the ties between the families. However, neither the bride nor the groom has significant input into the final decision [20]. Nepal is experiencing a slow shift away from arranged marriages to 'love' marriages, in which individuals have more freedom to choose whom they wish to marry, mostly with family approval [15,20].

Young women in Nepal lack decision-making power in matters related to sexuality, contraceptive use, and family size [21]. Communication between a young woman and her husband on matters related to sexuality is rare. Sex education in school and counselling services related to sex and sexuality are still taboo subjects. Moreover, many women in Nepal hold the view that it is in their *dharma *(defined as religion, moral duty and universal law) to be obedient, respectful, and pleasing to their husbands [23,24].

It is common knowledge that SVWM exists in Nepal, but it has received little attention from researchers, policy makers and programme managers, until very recently. There are only three previous small-scale population-based studies conducted in Nepal that explored the frequency and causes of SVWM among young women. These studies found that the prevalence of SVWM ranged from 12% to 50% in Nepal. They also identified various factors, such as women's lack of autonomy, high economic dependency of women on their husbands, men's perceived entitlement to force sex, lack of education and knowledge of sexuality, marriage practices, lack of family and legal support to women, and husband's use of alcohol, that contributed to sexual violence [14-16]. However, very little is known about how women define sexual violence, its negative consequences, and coping mechanisms used to avoid such situations.

Recognizing the existence of SVWM in Nepal, the Government passed a comprehensive law on gender-based violence in 2009 that made it a criminal offence for a husband to force his wife to have sex. The new law has a provision that includes fines and imprisonment for three to six months depending on the type of violence [25]. In practice, however, this law is not strictly enforced. Moreover, a large majority of Nepalese people including - local authorities, local police and other agencies that deal with gender-based violence - are still unaware of its existence [15].

The objective of this article is to contribute to the limited body of population-based research on sexual violence in developing countries, particularly during early years of marriage. First, we explore the perspectives of young women in Nepal on the definition of SVWM and the various forms it can take. Second, we examine the consequences of sexual violence and coping strategies reported by women. No previous study has examined these issues in Nepal, thus the results represent a first step toward understanding the problem of SVWM in order to inform an effective policy and programme response to prevent SVWM and to assist survivors of such violence.

## Methods

### Study area and population

Data for this paper was collected during a qualitative study entitled "Sexual violence among young couples in Nepal", carried out between 2006 and 2007. The study was conducted in two districts (Dang and Tanahu) among two major ethnic groups (Tharu and Brahmin/Chhetri) that inhabit the Terai (flat land/plains) and hill regions of Nepal. Comprising 28% of Nepal's total population, Brahmin/Chhetris are the largest ethnic group in Nepal and represent one of the more advantaged ethnic communities [20]. This group practices Hinduism and speaks Nepali, the national language of Nepal. The Tharus are the fourth largest ethnic group in Nepal (6.5% of the total population), and are one of oldest ethnic groups of the Terai belt [20]. Compared to Brahmin/Chhetri, Tharus are one of the most disadvantaged ethnic groups in Nepal. They also practices Hinduism, and farming and business are their main occupations [26]. The Dang and Tanahu districts were selected because they have a high concentration (more than 50% of the total population in the district) of the study's ethnic communities.

### Sample recruitment

After selecting the districts, a list of Village Development Committees (VDCs) that have a major concentration of the study's ethnic communities was prepared with the help of district-level key informants including a district development officer, heads of NGOs working in the health sector, the office-in-charge of district bureau of statistics, and leaders of political parties. One VDC per district was selected by applying simple random sampling to the list. A ward is the smallest administrative unit in Nepal and there are nine wards in a VDC. In this study, a ward or group of wards that had at least 100 households (at least 600 people) of the study's ethnic communities was considered to be a cluster and two clusters from each sampled VDC were selected by using simple random sampling.

After selecting clusters, an up-to-date list of households of the target ethnic communities was prepared with the help of local key informants (including: a village development secretary, village leaders, a school teacher, political party leaders, and social workers). A brief screening questionnaire was administered to the heads of the households. The screening questionnaire collected information on the number of family members usually living in the households, their sex, age, martial status and availability for interview, if eligible. The main purpose of the screening questionnaire was to identify eligible respondents for the study. Eligible respondents were defined as married women between the ages of 15 and 24 years. The screening questionnaire was administered to 123 heads of households and a list of potential participants was prepared. Although 142 eligible participants were identified in the screening, the free-listing (described in detail below) was conducted with 39 women aged 15-24 years (20 Tharu and 19 Brahmin/Chhetri) selected using simple random sampling from the list. The sample size for the free-listing was guided by the principle of information saturation that this study aimed to investigate (e.g. acts and behaviours that are considered sexual violence within marriage). Researchers have suggested that 20 to 30 respondents are sufficient to get a clear picture for multiple issues [27,28]. In cases where more than one eligible woman lived in a household, one woman per household was randomly selected to participate.

### Research tools and instruments

Free-listing is an open-ended interview technique in which informants are asked individually to list terms, such as local vocabularies, used for sexual violence [28]. In this study, free-listing was conducted to ascertain all acts and behaviours by husbands, as well as the perceived causes, which young women considered to be sexual violence. The following main questions were asked in the free-listing to understand women's perspectives on defining sexual violence within marriage and reasons for it:

• What are all the acts/behaviours of a husband to his wife that can be considered "sexual violence or sexual coercion"?

• Why does a young married woman experience sexual violence from her husband?

• Sometimes husbands physically force their wives to have sex against their wishes or desire. Have you ever had such a sexual experience? Could you please tell me about it?

In addition, 15 in-depth case histories were conducted with those women who had reported SVWM during the free-listing exercise. A detailed interview guide was used for in-depth interviews, which included the following main questions and probes:

• When two people marry or live together, they share both good and bad moments. In your relationship with your husband/partner, do you have any such personal experiences to share with me?

• Sometimes husbands touch their wives' bodies in a sexual way against their wishes or desire. Have you ever had such experiences? Could you please share such information with me?

• Sometimes husbands physically force their wives to have sexual intercourse against their wishes or desire. Do you have any such experiences to share?

• Why do you think that your husband acts/behaves in such way? Why would your husband force you to perform sexual acts you did not want to? Have you had any other undesired sexual experiences? Could you please explain further?

• In your opinion, what are the possible negative effects (consequences) of sexual violence on women's lives?

• Have you ever experienced such problems? What types of problems did you face/experience? When? Could you share them with me?

• What did you do to overcome such problems?

• In your opinion, how can a married woman avoid such undesired sexual experiences with their husbands?

• Have you used any approach to minimise or avoid such negative sexual experiences with your husband? What have you tried? Have you ever succeeded in using tactics to avoid undesired/negative sexual relations/experiences?

• Have you ever gone to a place or person for help and advice on these matters? When?

• If not, why didn't you go somewhere or contact someone?

Data was collected by two trained Nepali female research assistants (RAs). Interviews were conducted in a private location, convenient to the respondent, usually away from their homes. All case histories were tape-recorded. Training, supervision, and monitoring during the data collection were undertaken by the first two authors of this article to assure interview quality and respondents' privacy. None of the eligible respondents selected for the study refused to do an interview.

### Ethical considerations

The core protocol and research instruments were reviewed and approved by the Nepal Health Research Council and the World Health Organization's (WHO) Research Ethics Review Committees (ERC). Participants' verbal consent was obtained before their participation in the study. In Nepal, a request for written consent can have negative effects, as participants tend to be wary of any implied commitment resulting from signing a form. A consent form, written in the local language and using simple terms, was used for the screening questionnaire, free-listing, and case histories. It described the study objectives, nature of the participant's involvement, risk and benefits, and confidentiality of the data. This was read aloud to the individuals and their informed consent was obtained. Confidentiality of information was ensured by removing personal identifiers from the field notes and by securing access to all data and information. No cash incentives were provided to the respondents. However, all case history participants received a small gift (towel and soap) worth approximately four US dollars to compensate their time and transportation costs.

### Data analysis

The free-listing data were analysed by using the computer software ANTHROPAC, and percentage, average rank and Smith Salience are reported. The average rank refers to how early in the listing each person, on average, mentioned a particular response item. Therefore, the term that has the lowest rank was the term that was mentioned by most respondents first [29]. 'Smith's Salience' is an indicator that weights the frequency of mention with the average rank [29]. Therefore, if two items were mentioned an equal number of times, an item would still have higher salience if it was mentioned earlier in respondent's list.

The information obtained from the case histories was analysed using thematic analysis [28,30]. First, the case histories were transcribed from audio-tape by the field researchers and translated into English by two professional translators. After the authors reviewed the transcripts, the major themes were developed into codes for organising and analysing subsequent interviews. Two research assistants developed an initial code-book independently based on early interviews. Similarity and dissimilarity between two research assistants in assigning the codes in early interviews were checked by a third person (one of the authors of the paper), who resolved the discrepancies before coding the remaining interviews. Modifications to the code book were made in cases where the existing codes were not adequate and the updated code book was used in analysing subsequent interviews. Using Atlas.ti software, all the interviews were coded and linked with the background characteristics of the respondents. Once the transcripts were coded, relevant quotations that illustrated the emerging themes were integrated with the background characteristics of the participants in a single report. From these reports, the ranges of views expressed within the themes were explored. Finally, the relevant quotations were extracted and interpreted.

## Results

### Characteristics of the study population

Table 1 presents the selected socio-demographic characteristics of the young married women covered in the free-listing exercise. Approximately, a quarter of the women were between the ages of 15-19 years (mean age: 17.6 years). Most women were homemakers and had no independent cash income. One in three women had one child and 41% had two children. Approximately half of the participants had married before age 18. Roughly half of the women were either illiterate or only had primary level education. Only a quarter of the women (25.9%) had received 10 years or more education. Overall, Tharu women were less educated than Brahmin/Chhetri women. For example, 47% of Brahmin/Chhetri women (9 out of 19) had achieved 10 or more years of education compared to only 5% of Tharu women. Most husbands were literate, with greatest proportion involved in small business, followed by labour work, civil service, and agriculture driver, respectively.

**Table 1:** Selected socio-demographic background characteristics of young married women (15-24 years) included in the free-listing exercise

Background characteristics	N	Percent
**Current age (in years)**		
15-19	9	23.1
20-24	30	76.9
**Caste/Ethnicity**		
Tharu	20	51.3
Brahmin/Chhetri	19	48.7
**Level of education**		
No schooling	6	15.4
Primary and non-formal education	14	35.9
Secondary (6-10 grades)	9	23.1
SLC pass or above (More than 10 grades)	10	25.6
**Age at current marriage (in years)**		
Less than 18	20	51.3
18 or above	19	48.7
**Number of living children**		
None	8	20.5
One	13	33.3
Two	16	41.0
Three or more	2	5.1
**Women's main occupation**		
Homemaker	28	71.8
Agriculture	4	10.3
Business/small business	5	12.9
Services	1	2.6
Labourer	1	2.6
**Husband's level of education**		
No schooling	1	2.6
Primary and non-formal education	8	20.5
Secondary (6-10 grades)	13	33.3
SLC pass or above (More than 10 grades)	17	43.6
**Husband's main occupation**		
Business/small business	11	28.2
Labourer	9	23.1
Services	9	23.1
Unemployed	5	12.8
Agriculture	5	12.8
**Total**	**39**	**100.0**

Case histories were drawn from sub-samples of those who participated in the free-listing exercise. Therefore, their characteristics were very similar to those of the free-listing participants. For example, one-third of women (4 out of 15) were under 20 years of age. Most women were homemakers (10 out 15) and had no independent cash income. Over half of the women (8 out of 15) had at least one child. Over half of their husbands (8 out of 15) were involved in daily wage labour. Though information about the consumption of alcohol was not collected during the free-listing exercise, the case histories revealed that alcohol consumption was a more common and culturally acceptable behaviour among Tharu men and women than amongst Brahmin/Chhetri.

### Definition of sexual violence within marriage

During the free-listing exercise, participants were asked to list all acts and behaviours of their partners that they considered to be sexual violence. Interviews identified a total of 28 different acts and behaviours of sexual violence. Table 2 shows the 10 most frequently mentioned acts and behaviours defined by respondents as sexual violence.

**Table 2:** Acts and behaviours defined by young women as sexual violence within marriage: results from free-listing

Items (Nepali)	English translation	Frequency (n = 39)	Percent	Average rank	Smith's Salience Score*
*Ichha biparit yon samparka*	Sex against partner's wishes	29	74	2.724	0.540
*Bimari huda yon samparka*	Sex during illness	24	62	4.500	0.289
*Jaad raksi khayera yon samparka*	Forced sex after consuming alcohol	24	62	3.167	0.443
*Mahinawari huda yon samparka*	Sex during menstruation	21	54	3.381	0.352
*Yon samparka garna namane kutpit/gali/dhamki*	Physical or verbal abuse or threats following refusal of sex	21	54	4.143	0.303
*Sutkeri huda/bachcha sano huda yon samparka*	Sex after delivery/when baby is small	20	51	4.450	0.273
*Thakeko bela yon samparka*	Sex in spite of being tired	14	36	4.357	0.206
*Ichha biparit youn anga chalaune/stan samaune*	Unwanted sexual touch	11	28	5.273	0.121
*Aru sanga yon samparka*	Extramarital sexual relationship	8	21	4.375	0.121
*Jabarjasti yon samparka*	Forced sex	7	18	2.857	0.096

* Smith Salience score varies between 0-1. The most Salient (basic) terms of the domain have the value 1. The term not mentioned at all (least Salient) have the value 0.

Almost three-quarters (74%) of women designated 'sex against their wishes' as SVWM. Sixty-two percent of respondents mentioned non-consensual 'sex during illness' and 'forced sex after consumption of alcohol' as SVWM. Other acts and behaviours of a husband towards his wife which women considered to be sexual violence included sex during menstruation, physical/verbal torture/threats following refusal of sex, sex shortly after delivery/when the baby is small, sex when tired, and unwanted sexual touch.

The average rank shows that women were likely to mention 'forced sex' and 'forced sex despite verbal refusal' early in their listing of violent sexual acts by their husbands (rank 2.8 and 2.7). The Smith Salience score suggest that 'sex against the wife's wishes' was mentioned by a higher number of respondents and was mentioned earlier in the list than other acts and behaviours (Smith's Salience score 0.540).

### Types of SVWM

Although free-listing data do not aim to show the extent of sexual violence amongst the study population, it can indicate the scale of the problem. The free-listing data suggest that there was a high prevalence of SVWM in the study population. Approximately, half of the married women (19 out of 39) ever experienced SVWM. A higher percentage of Tharu women (55%) compared to Brahmin/Chhetri women (42%) reported forced sex. The type of SVWM ranged from verbal abuse to beating, and unwanted sexual touch to forced sex (Table 3).

**Table 3:** Number and percent of young women (15-24 years) ever-experiencing sexual violence by reported type of violence

Type of experience	n	Percent
Experienced unwanted physical touch	21	53.8
Experienced forced sex from husbands	19	48.7

Note: *N *= 39.

### Consequences of refusing sex

Case histories revealed that women who refused to have sex with their husbands suffered severe repercussions, such as physical and emotional abuse. Some of the consequences of refusing sex are as follows:

### Physical violence

Case histories revealed that more than half of the women were beaten (8 out of 15) if they denied their husbands for sex. Husbands used physical violence to force their wives to have sex, often when the husbands were drunk. Women also faced severe physical violence such as being beaten with an iron rod, pushed down the stairs and kicked in the abdomen during pregnancy. Women described their experiences as such:

"...I was often beaten and forced to have sex. He also beat me and forced me to have sex even when I was almost nine months pregnant. He is a shameless man. ...... Because of his beatings, my son died soon after his birth (with a sad face). But since I got married to him, I have to tolerate everything. He also forced me to have sex right after I gave birth to the child."

"...One night I was not feeling well and strongly denied sex. I said, "I am not going to let you have sex today, I am not feeling well." Then I turned my face to the other side but he got on top of me and forced me to have sex. I suddenly got up and tried to leave the room but he pulled my hair and started kicking my abdomen. He kicked me several times on my abdomen. I tried to stop him but I could not. I cried and begged him not to kick my abdomen but he continuously kicked till he cooled down (tears in her eyes)..."

### Emotional harassment

Some case histories (6 out of 15) revealed that husbands told their wives that they must have sex in order to prove their love. In some cases, the husband gave false information, stating that if the wife had sex during pregnancy, she would give birth to a son (a strong cultural incentive in Nepal). Another commonly reported method of coercion was for husbands to threaten taking a co-wife (9 out of 15). Although women reported that they often argue with their husbands, they felt that they have to submit eventually, even if unwillingly, as they have nowhere to go if their husband leaves them. For example:

"...He said, "if you do not let me have sex, I will go to other girls or I can even marry another woman!". He is my husband after all so I let him have sex with me ... he does not understand my feelings. All he wants is to fulfil his desire. Sometimes we argue a lot over this but I have no other alternatives."

### Accusation of infidelity

Most case histories (10 out of 15) revealed that scolding, abusing, and accusations of a wife's infidelity were some of the common tactics used by a husband to his wife for coerce sex. . One woman who was accused of an extra-marital relationship when she refused to have sex during menstruation said:

"One night he wanted to have sex with me. I denied him because I had my menses. He kept quiet that night but the next evening when he returned home drunk, he beat me badly. He called me a whore and asked me to get out of the house. He also accused me of sleeping with other men and that is why I refuse to have sex with him..."

### Health consequences

Most women (10 out 15) in the case histories reported that they experienced health problems after forced sex from their husbands. According to these women, problems such as backache, body ache, headache, and lower abdominal pain were commonly experienced after coerced sex. Some women (5 out of 15) also reported that they had experienced white discharge, vaginal itching, and dark blood flow. Due to cross-sectional nature of the study, it is difficult to ascertain the causal link between reported health problems by women and sexual violence. However, most of the women reported that white discharge, vaginal itching, lower abdominal pain, and bleeding occurred immediately after the violence. One woman explained:

*"Every time he forced me to have sex and beat me I wanted to die... Every time I have sex, dark blood flows from my vagina. I have been suffering from this problem for the last three month"*.

### Psychological consequences

Many women (11 out of 15) reported that they had experienced psychological trauma after they were coerced into having sex. Women reported being very depressed and stressed after coercive sexual experiences with their husbands.

"I get stressed very often. Sometimes I just could not sleep the whole night. I feel like crying all the time. I also cry often on my own. Sometimes I feel like I should leave him .... Once a woman gets married, she should compromise and tolerate her husband and obey him as if he is everything in her life."

"I have become absent-minded these days. Sometimes, I feel like I should commit suicide. I feel very bad as everyone in the community knows about me. I feel like crying all the time. Sometimes I cannot sleep the entire night. I also do not like to eat. But I do not know how long I will have to tolerate it"

### Coping strategies

Data from the study suggest that most women (10 out of 15) adopted strategies to avoid situations that place them at risk of sexual violence from their husbands. However, they could not protect themselves from being sexually coerced despite the strategies they used. Some of the strategies employed by women are as follows:

### Physical resistance

Ten out of 15 women reported that they made noises or screamed when their husbands forced sex on them. However, it was observed that other family members or neighbours did not intervene as they consider it a family matter. Nine of the 15 women stated that they fought back against their husbands in order to protect themselves. One woman shared her experience:

*"As usual, he came home drunk and wanted to have sex. I slept turning my face to the other side. He started scolding me saying "I don't care whether you have desire or not, you have to sleep with me, otherwise, you get out of my house". He forcefully turned me over to him, but I suddenly got up and tried to escape from the bed. But he slapped me badly on my cheek. I also punched him and pushed him away. Then he pulled my hair and threw me on the floor"*.

### Awaking children or sleeping separately

Women who had small children (6 out of 15) attempted to avoid violence by waking up or holding their children. Women frequently stated that if they knew or suspected that the husband was going to force sex, she would hold the child while sleeping in order to protect herself from potential abuse. They also mentioned that even if their children were sleeping they would wake them up and sleep with them. For example:

*"I often sleep turning to the other side. I also make my body very tight so that he cannot move it. I always use a different blanket and roll inside it so that he cannot touch me. One day, as usual, he was drunk and tried to force me to have sex. At that time, I held my son in my arms so tightly and got up from the bed. He could not beat me because of my son. He tried to pull my son but I screamed and my son started crying so he could not touch me. Then I unlocked the door and ran next door"*.

### Pretending to be ill/feigning menstruation and visiting maternal home

In the case histories, 7 out of 15 women also reported that sometimes they pretended to be ill or feigned menstruation to avoid sexual violence from their husbands. For example:

"I make some excuses like, 'I have stomach pain or I am pregnant' or having menstruation"

Four out of 15 women also reported that sometimes they visit their maternal home and stay for few days to save themselves from sexual violence.

### Care and support seeking behaviour

Eleven of the 15 women reported that they felt socially isolated and generally did not turn to institutions, families, or friends for advice and support. Women considered it shameful to share such personal problems with others. Many women (11 out of 15) reported thinking that they are the only ones who face such violence and therefore do not talk about their problems, as they perceive that they will receive no support from family or friends. The case histories revealed that only about half the women (7 out of 15) had ever told anyone about their problems. These women had either told their mothers, mothers-in-law, close friends, or a neighbour. None of the women, however, had sought help from organisations or health providers. For some women, the first time they shared their experiences of sexual violence was in the interviews.

## Discussion

Results from this study suggest that SVWM is common amongst young married women in Nepal. The extent of sexual violence may vary according to the definition used; however, we found that one in two women reported having experienced some forms of SVWM, as defined in this study. The estimated prevalence from this study is quite high when compared to neighbouring countries such as India and Bangladesh [13,17,31]. A higher percentage of Tharu women (55%) reported experiencing SVWM than Brahmin/Chhetri women (42%). This may be due to the lower levels of education and higher levels of alcohol consumption among Tharu than amongst Brahmin/Chhetri men and women.

The case histories suggest that reported sexual violence experiences may represent only the tip of the iceberg because of underreporting due to the stigma and shame linked to having experienced such behaviours from an intimate partner. The suffering of the victims of sexual violence and those who refuse sex remain largely unreported.

Women reported symptoms such as backache, body ache, headache, lower abdominal pain, vaginal bleeding and thoughts of suicide suicidal ideation immediately after the assault. Psychological trauma and depression following a coercive sexual experience was reported by young married women in Nepal. These consequences were not reported in other studies from South Asia. Due to the exploratory nature of this study, a causal link between sexual violence and physical and psychological health problems reported by women cannot be established. Nevertheless, the findings provide circumstantial evidence of links between sexual violence and the negative physical and psychological health consequences and are consistent with previous research [7,32].

Results demonstrated that young Nepali women use various coping strategies to avoid situations that place them at risk of sexual violence from their husbands. Some of these strategies are similar to those reported by women in Bangladesh [13,17]. However, 'fighting back' against their husbands and visiting the maternal home to escape SVWM were findings unique to this study. However, in most cases, women reported not being able to protect themselves from SVWM. Young women who suffer from SVWM appear to be isolated and lack support options. Therefore, developing a comprehensive health sector response to the various impacts of SVWM is vital.

There are three main limitations of this study. First, underreporting associated with respondents' reluctance to report highly sensitive experiences may have produced an underestimate of the prevalence of SVWM in our sample. Second, due to the qualitative nature of the study, our analysis should be regarded as exploratory and the findings as suggestive. Third, the cross-sectional nature of the data limits our ability to establish temporality or causality in many of the observed relationships. It is possible that the relationships work in the reverse direction or that the outcomes are caused by unmeasured variables.

These limitations notwithstanding, this article makes an important contribution to the literature on SVWM in developing countries. First, it clarifies Nepali women's understanding of the meaning of sexual violence that is essential to defining potential causes and consequences of sexual violence. Second, it provides further evidence on the link between sexual violence and women's health. Third, it generates information on the care and support seeking behaviour of young married women who experience SVWM that is required in developing effective policies to address the problem and in designing appropriate programme responses. While additional research is helpful in documenting the level of SVWM, the evidence provided in this paper calls for immediate actions to prevent SVWM amongst young married women and to manage its consequences.

## Conclusions

The recognition of the pervasiveness of SVWM amongst young married Nepali women, as well as, the serious health and psychological consequences that results from it, is essential in dealing with the problem. Health care providers need to be sensitive to signs of SVWM in order to effectively manage it's consequences and to provide referrals, as needed. Policy-makers should promote prevention of SVWM and health officials should provide services to women who have experienced intimate partner violence. In particular, it is important to address the demonstrated reluctance of abused women to seek help. Health providers should inquire about SVWM during routine visits by women. At the service level, a comprehensive response is needed to tackle SVWM. All health care services should promote the prevention of SVWM and provide care to victims. It is necessary to improve access to, and reduce the stigma of mental health services for women suffering from mental health consequences of SVWM, particularly thoughts of suicide. Moreover, reproductive health providers should be sensitized and trained to recognize and respond to SVWM. Community leaders and NGOs can be effective in prevention of SVWM and in promoting care-seeking amongst the victims.

Finally, further research is needed to understand the scale and determinants of SVWM amongst young couples in Nepal. Such research should aim to deepen understanding of both the risk and protective factors related to violence, focussing particularly on identifying interventions to prevent and manage SVWM. Longitudinal research is also needed to better establish the consequences of SVWM. Research on male attitudes and beliefs that contribute to intimate partner violence is also needed to gain a comprehensive understanding of the problem.

This paper represents a much needed first step towards defining sexual violence within the early years of marriage from the perspectives of young married women in Nepal. The paper also informs us of the need for an effective policy and programme response to assist survivors of such violence. In addition, we hope that our study contributes to the limited body of population-based evidence on SVWM in developing countries.

## Competing interests

The authors declare that they have no competing interests.

## Authors' contributions

MP conceived and designed the study. He monitored and supervised the data collection, analysed the data, and prepared the manuscript. JT developed study instruments, and was involved in data analysis and preparing the manuscript. IS reviewed the paper and contributed to revising it critically for intellectual content. All authors read and approved the final content of the manuscript.

## Pre-publication history

The pre-publication history for this paper can be accessed here:

http://www.biomedcentral.com/1471-2458/11/29/prepub

## References

[B1] World Health Organisation World Report on Violence and Health 2002 World Health Organization, Geneva

[B2] Koenig M Zabolkota I Lutalo T Nalugoda F Wagmen J Gray R Coerced first intercourse and reproductive health among adolescent women in Rakai, Uganda International Family Planning Perspectives 2004 20 4 156 163 10.1363/3015604 15590381

[B3] Kishor S Johnson M Profiling domestic Violence: a Multi-Country Study 2001 Macro International, Calverton, MD, USA

[B4] Parish WL Wang T Laumann EO Pan S Luo Y Intimate partner violence in China: National prevalence, risk factors and associated health problems International Family Planning Perspectives 2004 30 4 174 81 15590383 10.1363/3017404

[B5] Martin SL Tsu AO Maitra Singh K Kupper LL Sexual behaviours and reproductive health outcomes: associations with wife abuse in India The Journal of the American Medical Association 1999 28 20 1967 72 10.1001/jama.282.20.1967 10580466

[B6] Caceres CF Marin BV Hudes ES Sexual coercion among youth and young adults in Lima, Peru Journal of Adolescent Health 2000 27 5 361 367 10.1016/S1054-139X(00)00096-3 11044709

[B7] World Health Organisation WHO Multi-Country Study on Women's Health and Domestic Violence against Women 2005 World Health Organization, Geneva

[B8] Zierler S Feingold L Laufer D Adult survivors of childhood sexual abuse and subsequent risk of HIV infection American Journal of Public Health 1991 81 5 572 575 10.2105/AJPH.81.5.572 2014856 PMC1405069

[B9] Garcia-Moreno C Watts C Violence against women: its importance for HIV/AIDS prevention AIDS 2000 14 suppl.3 253 265 11086869

[B10] Maman S Intersection of HIV and violence: direction for future research and interventions Social Science & Medicine 2000 50 4 459 478 10.1016/s0277-9536(99)00270-1 10641800

[B11] Watts C Mayhew S Reproductive health Services and intimate partner violence; Shaping a pragmatic response in Sub-Saharan Africa International Family Planning Perspectives 2004 30 40 207 213 15590387 10.1363/3020704

[B12] World Health Organisation/London School of Hygiene and Tropical Medicine Preventing intimate partner and sexual violence against women: taking action and generating evidence 2010 Geneva, World Health Organisation

[B13] Santhya KG Jejeebhoy S S. Jejeebhoy, I. Shah & S. Thpa Young women's experiences of forced sex within marriage: evidence from India Sex with consent: Young people in developing countries 2005 New York: Zed Books

[B14] Puri M Cleland J Assessing the factors sexual harassment among young female migrant workers in Nepal Journal of Interpersonal Violence 2007 22 11 1363 1381 10.1177/0886260507305524 17925287

[B15] Puri M Shah I Tamang J Exploring the nature and reasons associated with sexual violence within marriage among young women in Nepal Journal of Interpersonal Violence 2010 25 10 1873 1892 10.1177/0886260509354514 20139347

[B16] Women's Rehabilitation Centre (WOREC) Breaking the Silence: Needs Identification of Victims of Gender-based Violence 2002 Kathmandu, Nepal

[B17] Khan ME Townsend JW D'Costa S Behind closed doors: A qualitative study on sexual behaviour of married women in Bangladesh Culture, Health & Sexuality 2002 4 2 237 256

[B18] George A Embodying identity through heterosexual sexuality-newly married adolescent women in India Culture, Health & Sexuality 2002 4 2 207 222

[B19] Joshi AM Dhapolam E Experiences and perceptions of marital sexual relationships among rural women in Gujrat, India Asia-Pacific Population Journal 2001 16 2 177 194

[B20] Central Bureau of Statistics Population Monograph of Nepal: Central Bureau of Statistics, National Planning Commission, Government of Nepal 2003 Kathmandu, Nepal

[B21] Puri M Unintended pregnancy among young couples in Nepal: Determinants and consequences of unintended pregnancy 2009 VDM Verlag Dr. Muller Aktiengesellschaft & Co. KG, Germany

[B22] Ministry of Health [Nepal], New Era, and ORC Macro Nepal Demographic and Health Survey Nepal Demographic and Health Survey 2006 2006 Calverton, Maryland USA: Family Health Division, Ministry of Health; New Era; and ORC Macro

[B23] Bennett L Dangerous Wives and Sacred Sisters. Social and symbolic Roles of High caste women in Nepal 1983 Columbia University Press, New York

[B24] Cameron M On the edge of the auspicious: Gender and Caste in Nepal 2005 University of Illinois Press, Chicago and Mandala Publications, Kathmandu

[B25] Government of Nepal The Domestic Violence and Punishment Act 2065 2009 Kathmandu, Nepal Government of Nepal

[B26] McDonaugh C Aspects of social and cultural change in a Tharu village community in Dang, west Nepal Nepal Tharu and Tarai neighbours 1999 HO Skar. Bibliotecha

[B27] Weller SC Romney AK Systematic Data Collection 1988 Thousand Oaks, CA: Sage

[B28] Campbell O Cleland J Collumbein M Southwick K Social science methods for research on reproductive health 1999 Geneva: World Health Organization

[B29] Borgatti SP ANTHROPAC 4.0. Methods guide 1996 Analytic Technologies. Natic

[B30] Ulin PR Robinson ET Tolley EE McNeill ET Qualitative Methods: A field guide for applied research in sexual and reproductive health 2002 Family Heath International, Research Triangle Park, USA

[B31] Koenig MS Ahemed M Hossain Mozumder ABM Women's status and domestic violence in Rural Bangladesh: Individual-and-community level effect Demography 2003 40 2 269 288 10.1353/dem.2003.0014 12846132

[B32] Heise L Toubia N Sexual coercion and reproductive health: A Focus on Research 1995 The Population Council, New York

